# An interpretable machine learning model for predicting central lymph node metastasis in cN0 T1–T2 papillary thyroid carcinoma: a retrospective study

**DOI:** 10.3389/fendo.2026.1803663

**Published:** 2026-04-27

**Authors:** Yalin Zhu, Ying Che, Shuhang Gao, Lina Wang, Shuangsong Ren

**Affiliations:** Department of Ultrasound, The First Affiliated Hospital of Dalian Medical University, Dalian, China

**Keywords:** central lymph node metastasis (CLNM), inflammatory indices, machine learning (ML), papillary thyroid cancer (PTC), prediction model, SHAP, thyroid function indices

## Abstract

**Introduction:**

This study aimed to develop and validate an interpretable machine learning model for preoperative prediction of central lymph node metastasis (CLNM) in patients with clinically node-negative (cN0) T1–T2 papillary thyroid carcinoma (PTC).

**Methods:**

A retrospective analysis was conducted on 710 patients (971 lesions), integrating pathological, ultrasound features, thyroid function, and systemic inflammatory indicators. A hierarchical feature selection strategy combining L2 and LASSO regularization was employed to optimize multimodal predictors and reduce overfitting. An explainable gradient boosting decision tree (GBDT) model was constructed and evaluated using calibration curves, decision curve analysis, and SHAP interpretability frameworks.

**Results:**

The model identified five independent predictors of CLNM: bilateral laterality, tumor size >1.0 cm, age ≤55 years, systemic immune-inflammation index (SII) >449.85, and platelet-to-lymphocyte ratio (PLR) ≤134.88; free triiodothyronine (FT3) was also included as an adjunct variable to enhance performance. The model achieved an AUC of 0.830 (95% CI: 0.773–0.887) in the test set, with robust performance confirmed after correcting for sample overlap (AUC 0.812) and external validation on an independent temporal cohort (n=50, AUC 0.800). The model showed clinical utility across a wide decision threshold range (0–85%).

**Discussion:**

This multimodal, interpretable prediction tool provides a non-invasive and clinically transparent aid for individualized surgical decision-making in cN0 T1–T2 PTC, bridging endocrine and inflammatory biomarkers with machine learning to advance precision thyroid oncology.

## 1 Introduction

Based on global cancer statistics for 2020 (GLOBOCAN2020), Thyroid cancer accounts for 586, 000 cases worldwide, ranking ninth in incidence in 2020 (1). The increase in thyroid cancer incidence is almost entirely due to the increase in Papillary thyroid carcinoma (PTC), It accounts for 80-85% of all thyroid cancers (2). While PTC is generally associated with an excellent prognosis and a high 10-year survival rate, 30% to 80% of PTC patients have the risk of early lymph node metastasis (LNM). Among them, the incidences of central lymph node metastasis (CLNM), lateral lymph node metastasis (LLNM), and mediastinal metastasis are 56.6%, 34.1%, and 9.3% respectively, even in those with clinically node-negative (cN0) disease. Lymph node metastasis is considered a significant prognostic factor influencing both disease recurrence and patient survival (3–5). The management of the central lymph node compartment (level VI) remains a pivotal aspect of surgical treatment. Prophylactic central lymph node dissection (pCLND) in cN0 patients has been a subject of long-standing debate. Supporters believe that pCLND can achieve accurate staging, guide subsequent treatment decisions (such as radioactive iodine ablation), and potentially reduce the risk of local recurrence. However, opponents argue that this surgical method also entails an increase in a series of complications, such as hypoparathyroidism and injury to the recurrent laryngeal nerve, which can significantly affect the quality of life of patients. But there is currently no clear evidence to suggest that this surgical method can bring overall survival benefits to low-risk patients (6, 7). Therefore, there is an urgent need for methods with high sensitivity and accuracy to predict CLNM, providing a theoretical basis for the clinical selection of appropriate management strategies.

Currently, the main method for preoperative identification of CLNM is ultrasound examination. Ultrasound has a high value in evaluating lateral cervical lymph node metastasis (LLNM), with a sensitivity exceeding 70%. However, the diagnosis of central lymph node metastasis (CLNM) has a sensitivity lower than 30% (8), because the central lymph nodes are affected by their anatomical location, the obstruction of the sternum, and the interference of gas in the trachea, the diagnostic value of ultrasound assessment is limited (9). The sensitivity of contrast-enhanced CT is relatively low, ranging from 41% to 66.7% (10, 11). The high incidence of CLNM and the low sensitivity of US and CT make it challenging to determine which factors are associated with subclinical CLNM.

Inflammation can promote tumor tissue proliferation, angiogenesis, and metastasis through multiple mechanisms and pathways (12). Thyroid cancer is closely related to inflammation, and the lymphocyte-associated inflammation index is closely related to the development of thyroid cancer (3). Commonly used clinical indicators of inflammation, including the neutrophil -lymphocyte ratio (NLR), lymphocyte-monocyte ratio (LMR), plateletly-mphocyte ratio (PLR), systemic inflammation index (SII) and systemic inflammation Response Index (SIRI). In thyroid cancer, systemic inflammatory indices such as NLR, PLR, SII and SIRI have shown prognostic value and correlate with aggressive clinicopathological features (3, 10–12). However, the integration of inflammatory markers with other clinical and imaging parameters for CLNM prediction remains underexplored.

Therefore, there is an urgent need to develop and validate robust preoperative predictive models that integrate clinical, serological, and imaging characteristics to reliably stratify the risk of CLNM in PTC patients. This study aims to identify the key predictive factors and construct a predictive model to facilitate individualized preoperative risk assessment, thereby guiding the most appropriate surgical management and improving patient prognosis.

## 2 Materials and methods

### 2.1 Study design and participants

Patients: The research was conducted in accordance with the Helsinki Declaration and was approved by the Ethics Committee of the First Affiliated Hospital of Dalian Medical University (document number: PJ-KS-KY-2024-542; and registered in the Chinese Clinical Trial Registry, registration number: ChiCTR2400090597); since it is a retrospective design, informed consent forms were not required to be signed. We conducted a single-center retrospective diagnostic-prediction study to develop and internally validate models for predicting central compartment (levels VI/VII) lymph node metastasis (CLNM) in differentiated thyroid carcinoma (DTC). Reporting follows TRIPOD (prediction model development and internal validation). We collected clinical data of 710 patients who underwent partial or total thyroidectomy and central lymph node dissection at the First Affiliated Hospital of Dalian Medical University from January 1, 2020 to June 1, 2024.

#### 2.1.1 Inclusion criteria

(1) T1–T2 classification according to the 8th Edition of the AJCC TNM Staging System (T1: tumor diameter ≤20 mm; T2: tumor diameter >20 mm but ≤40 mm; thus maximum tumor diameter ≤40 mm); (2) cN0 (no suspected lymph node metastasis on preoperative imaging); (3) postoperative pathological diagnosis of PTC; (4) underwent unilateral lobectomy with isthmusectomy or total thyroidectomy with central lymph node dissection, with pathological confirmation of central lymph node status.

#### 2.1.2 Exclusion criteria

(1) The diagnosis of post-operative paraffin-embedded tissue was pathologically confirmed as non-PTC or mixed PTC; (2) no central lymph node dissection was performed during the operation; (3) evidence of hematological diseases; (4) evidence of autoimmune diseases; (5) evidence of acute or chronic inflammation before surgery or other diseases that may affect routine blood tests; (6) Pathological or clinical diagnosis of distant metastasis; (7) incomplete clinical information.

**Figure 1:**
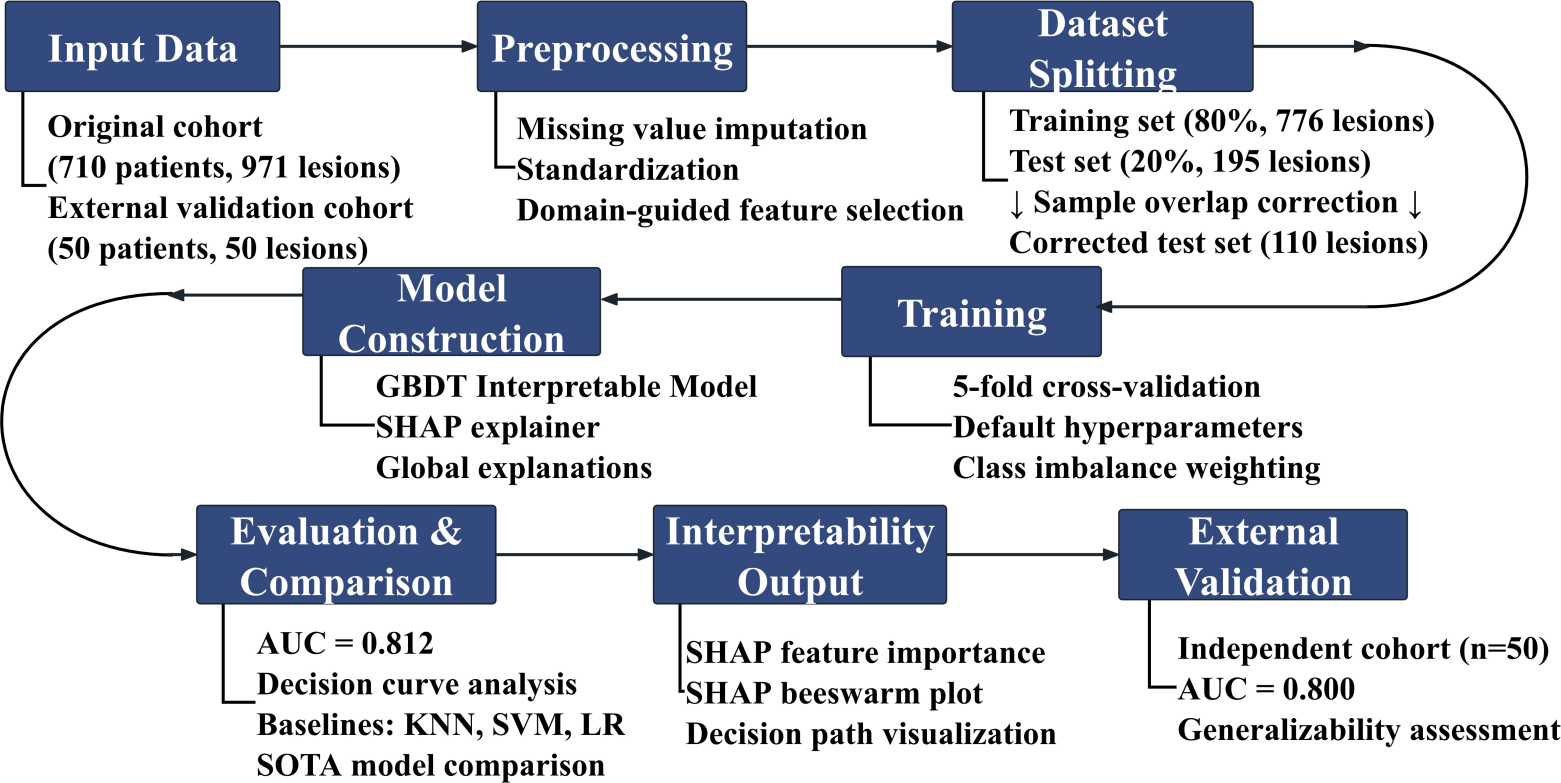
Schematic workflow of the interpretable GBDT model for predicting central lymph node metastasis (CLNM) in thyroid cancer.

The overall workflow is illustrated in **Figure 1**.

The study enrolled a primary cohort of 710 patients with 971 thyroid lesions and an independent external validation cohort of 50 patients with 50 lesions. After preprocessing (including missing value imputation, feature standardization, and domain-guided feature selection), the primary cohort was split into an 80% training set (776 lesions) and a 20% test set (195 lesions), with sample overlap correction to obtain a final independent test set of 110 lesions. A gradient boosting decision tree (GBDT) model was trained with 5-fold cross-validation, default hyperparameters, and class imbalance weighting. SHAP analysis was used for model interpretability (global feature importance bar chart and beeswarm plot). Model discrimination was evaluated in the internal test set (AUC = 0.812) via decision curve analysis and compared with baseline models (KNN, SVM, LR) and state-of-the-art (SOTA) models. The model was further validated in the independent external cohort (AUC = 0.800) to confirm its generalizability.

### 2.2 Prediction unit

The primary prediction unit was the individual lesion. In patients with multifocal PTC, it is clinically impossible to determine preoperatively which nodule will metastasize, and postoperative pathology cannot attribute a metastatic node to a specific lesion. Thus, the outcome (CLNM status) is shared across all lesions from the same patient. Lesion-level analysis integrates nodule-specific ultrasound features with patient-level data for granular risk assessment. Accordingly, all 971 lesions from 710 patients were treated as independent units.

This study introduces several methodological innovations to overcome the limitations of existing prediction models. First, we propose a domain-guided feature selection strategy. This strategy screens features within each clinical domain before cross-domain integration. It preserves clinical specificity and overcomes the limitations of conventional black-box feature aggregation. Second, we implement rigorous sample overlap correction. We identify and remove overlapping lesions between training and test sets, a critical issue often overlooked in lesion-based predictive modeling. Third, we provide comprehensive model interpretability using SHAP (SHapley Additive exPlanations) analysis. This enables both global feature importance assessment and individualized prediction explanation. Fourth, we conduct multiple robust sensitivity analyses to validate model generalizability across different clinical scenarios.

### 2.3 Outcome and predictor domains

CLNM was determined on surgical pathology for level VI and upper level VII nodes based on standard anatomic boundaries.

*A priori* predictor domains. Candidate variables were categorized into four clinical domains. To ensure unbiased model development, only preoperative variables were used as predictors; postoperative variables (e.g., capsular invasion, CLNM status) served solely as the outcome reference standard.

- Pathological features (preoperative only): Preoperative FNA cytology was reported per the Bethesda classification. Intraoperative and postoperative findings (e.g., capsular invasion, CLNM) were used exclusively as the outcome reference standard and excluded from predictors to ensure rigorous model development.
- Ultrasound characters: Tumors (composition, echo, shape, margin, and calcification status), as well as the location, number, and lateral orientation of the tumors. According to the American College of Radiology Thyroid Imaging Reporting and Data System (ACR TI-RADS), the primary lesion was evaluated as follows: echo (hypoecho indicates low or extremely low echo; other types indicate no echo, high echo, or equal echo); shape (long-to-short ratio > 1; long-to-short ratio < 1); margin (clear indicates smooth); composition (cystic indicates cystic or spongy; solid indicates mixed cystic-solid, solid); and calcification (negative indicates no or presence of large comet-tail-like artifacts; positive indicates coarse calcification, peripheral calcification, or punctate strong echo).
- Thyroid function indicators: Thyroid−stimulating hormone (TSH, reference range: 0.35–5.10 μIU/L), free triiodothyronine (FT3, reference range: 2.76–6.45 pmol/L), free thyroxine (FT4, reference range: 11.2–23.81 pmol/L), thyroglobulin (Tg, reference range: 3.5–77.00 ng/ml; negative: ≤ 77.00 ng/ml, positive: > 77.00 ng/ml), anti−thyroglobulin antibody (TgAb, reference range: 0.00–115.00 IU/mL; negative: ≤ 115.00 IU/mL, positive: > 115.00 IU/mL), anti−thyroid peroxidase antibody (TPOAb, reference range: 0.00–34.00 IU/ml; negative: ≤ 34.00 IU/ml, positive: > 34.00 IU/ml), and thyrotropin receptor antibody (TRAb, reference range: 0.00–1.75 IU/L; negative: ≤ 1.75 IU/L, positive: > 1.75 IU/L).
- Inflammation indicators: The following inflammatory indices were evaluated: neutrophil−to−lymphocyte ratio (NLR), platelet−to−lymphocyte ratio (PLR), lymphocyte−to−monocyte ratio (LMR), systemic immune−inflammation index (SII = Neutrophil count × Platelet count ÷ Lymphocyte count), and systemic inflammatory response index (SIRI = Neutrophil count × Monocyte count ÷ Lymphocyte count).
- Cut−off determination and grouping: The optimal cut−off value for each indicator in predicting CLNM was determined using receiver operating characteristic (ROC) curve analysis. Patients were then categorized into corresponding groups based on these cut−offs.

Neck ultrasound was performed by board−certified sonographers using high−frequency linear probes following a standardized reporting template. Preoperative laboratory tests were conducted on accredited analytical platforms according to the manufacturers’ instructions. Fine−needle aspiration (FNA) was carried out when clinically indicated; results from nodal FNA were excluded from the predictive variables to avoid incorporation bias. Patient demographics (age, gender) and tumor characteristics (laterality, multifocality) were collected from medical records. Postoperative findings (capsular invasion, CLNM status) were obtained from pathology reports and served exclusively as the outcome reference standard (CLNM-negative vs. CLNM-positive), not as predictors.

### 2.4 Data preprocessing

Continuous variables were inspected for plausibility; extreme outliers were winsorized at the 1st/99th percentiles when clinically implausible. Categorical predictors were one-hot encoded. Variables with ≤20% missingness were imputed within training folds (median for continuous; mode for categorical) with parameters applied to test data; variables with >30% missingness in any domain were excluded from that domain. All continuous predictors were z-standardized within the derivation data. Pairwise collinearity was screened (Pearson |r|>0.8); one of each collinear pair was removed based on clinical interpretability. Derived inflammatory indices (NLR, PLR, LMR, SII, SIRI) were computed from the same blood draw.

### 2.5 Experimental workflow

We pre-specified five experiments to systematically evaluate feature importance and model performance. The first four trained domain-specific models to screen and rank features within each clinical domain; the fifth combined information across domains. This hierarchical design ensures that feature selection is clinically interpretable and avoids the limitations of black-box feature aggregation.

- Experiment 1: Pathology-related model (feature screening).
- Experiment 2: Ultrasound model (feature screening).
- Experiment 3: Thyroid-function model (feature screening).
- Experiment 4: Inflammation model (feature screening).
- Experiment 5: Combined modeling across domains.

Within Experiments 1–4, we fit L2-penalized logistic regression (standardized inputs). Feature importance was defined as the absolute standardized coefficient (|β|), yielding a within-domain ranking from highest to lowest weight.

### 2.6 Baseline clinical model

We developed a simple baseline model using multivariable logistic regression with age, tumor size, and laterality. This model was trained and validated using the same data and procedures as the combined model.

### 2.7 Combined modeling strategies

Top-features merge with LASSO gatekeeping. We carried forward the top three features by |β| from each domain (≤12 predictors). An L1-penalized logistic regression (LASSO) with nested cross-validation (inner 5-fold; 1-SE rule) selected a parsimonious subset. We then refit an unpenalized multivariable logistic regression on the retained features to estimate adjusted effects and obtain final scores.

### 2.8 Gradient boosting decision tree (GBDT) model

In addition to the logistic regression-based model, we developed a GBDT model using the six selected features (age, tumor size, laterality, FT3, PLR, and SII). GBDT is an ensemble learning method that builds a series of decision trees sequentially, where each new tree corrects the errors of the previous trees. The final prediction is the sum of the contributions from all trees. We used the default hyperparameters as implemented in scikit-learn (n_estimators = 100, learning_rate = 0.1, max_depth = 3). The GBDT model was trained on the training set and evaluated on the corrected test set.

### 2.9 Training, validation, and performance metrics

The dataset was split into 80% derivation and 20% hold-out test sets, stratified by CLNM. During initial data partitioning, 85 lesions from patients with multifocal tumors were inadvertently assigned to both the training and test sets. This occurred because lesions from the same patient were split across sets. To correct this, we removed these 85 overlapping lesions from the test set. The final testing set comprised 110 lesions with no overlap with the training set (776 lesions). All subsequent model evaluations used this corrected testing set. This correction is essential for rigorous model evaluation and represents a key methodological contribution of our study.

Model building (including imputation, scaling, selection, and hyperparameters) used repeated stratified 5-fold cross-validation (10 repeats) within the derivation set. Class imbalance, if present, was addressed via inverse class-frequency weighting.

### 2.10 External validation

In addition, an independent external cohort of 50 patients was collected from the same institution during a different time period (July–September 2024) to assess model generalizability. The same inclusion and exclusion criteria were applied as for the primary cohort. The model was applied to this cohort without any retraining, using the same preprocessing parameters (e.g., standardization mean and standard deviation) derived from the training set. This cohort was used solely for external validation and was not involved in any model development steps.

### 2.11 Interpretability and feature ranking

For each domain, we report the feature ranking from highest to lowest |β|. We also provide the pre-LASSO ranking and the post-LASSO retained set with adjusted odds ratios (ORs) and 95% CIs. To enhance clinical transparency, we employed SHAP analysis to interpret the final GBDT model. SHAP values quantify each feature’s contribution by calculating its marginal contribution across all possible feature subsets. We generated SHAP summary dot plots to visualize global feature importance and the direction of feature effects. We also generated SHAP bar plots to rank features by mean absolute SHAP values. These visualizations address the “black-box” limitation of machine learning models. They help clinicians understand which features drive predictions and how feature values influence individual predictions. As a sensitivity check for functional form, we probed key continuous predictors using restricted cubic splines. If nonlinearity improved the Akaike Information Criterion (AIC), we retained spline terms in a sensitivity model while preserving the selection pipeline.

### 2.12 Sensitivity and robustness analyses

To assess model robustness across different conditions, we conducted the following pre-specified sensitivity analyses, each designed to test a specific methodological assumption:

(1) Feature selection stability: We varied the number of top features selected per domain (K = 1, 2, 3, 4) to evaluate whether model performance depends on this hyperparameter.

(2) Class imbalance handling: We compared inverse class-frequency weighting with minority-class bootstrap upsampling to assess the impact of imbalance correction methods.

(3) Missing data handling: We performed complete-case analysis as an alternative to the primary multiple imputation approach to verify that missing data imputation did not bias results.

(4) Measurement interval sensitivity: We excluded patients with intervals greater than 14 days between ultrasound and laboratory testing to assess whether measurement timing affects model performance.

(5) Patient-level clustering effect: To address the potential non-independence of multiple lesions from the same patient, we constructed an independent patient-level test set of 100 solitary-lesion patients (49 CLNM-positive, 51 negative) and re-evaluated model performance. This analysis tests whether within-patient clustering materially biases lesion-level model estimates.

Model robustness was judged by comparing changes (Δ) in AUC, calibration slope, and decision curve net benefit across these sensitivity analyses.

### 2.13 Statistical methods

To ensure unbiased model development, only preoperative variables were used in model development; postoperative variables served solely as the outcome reference standard.

Optimal cutoffs for inflammatory indices (NLR, PLR, LMR, SII, SIRI) were determined by maximizing the Youden index based on ROC analysis in the training cohort. These cutoffs were used only for descriptive purposes and subgroup analyses; all continuous variables were entered as continuous variables in model development.

Six machine learning algorithms (K-nearest neighbors, logistic regression, support vector machine, decision tree, gradient boosting machine, and random forest) were implemented using default hyperparameters in scikit-learn. No hyperparameter tuning was performed.

#### 2.13.1 Model performance evaluation

Discrimination was assessed using the area under the ROC curve (AUC) with 95% confidence intervals (2, 000 bootstrap samples). Calibration was evaluated using calibration slope, intercept, and Brier score. Clinical utility was assessed using decision curve analysis (DCA). All analyses were performed using R (glmnet, pROC, rms, rmda) and Python (scikit-learn). Random seeds were fixed for reproducibility.

#### 2.13.2 Baseline comparisons

SPSSAU (Version 24.0) was used for baseline comparisons. Categorical data were compared using chi-squared or Fisher’s exact tests. Normally distributed continuous data were compared using t-tests, and non-normally distributed data using Mann-Whitney U tests.

## 3 Results

### 3.1 Patient cohorts

**Figure 2:**
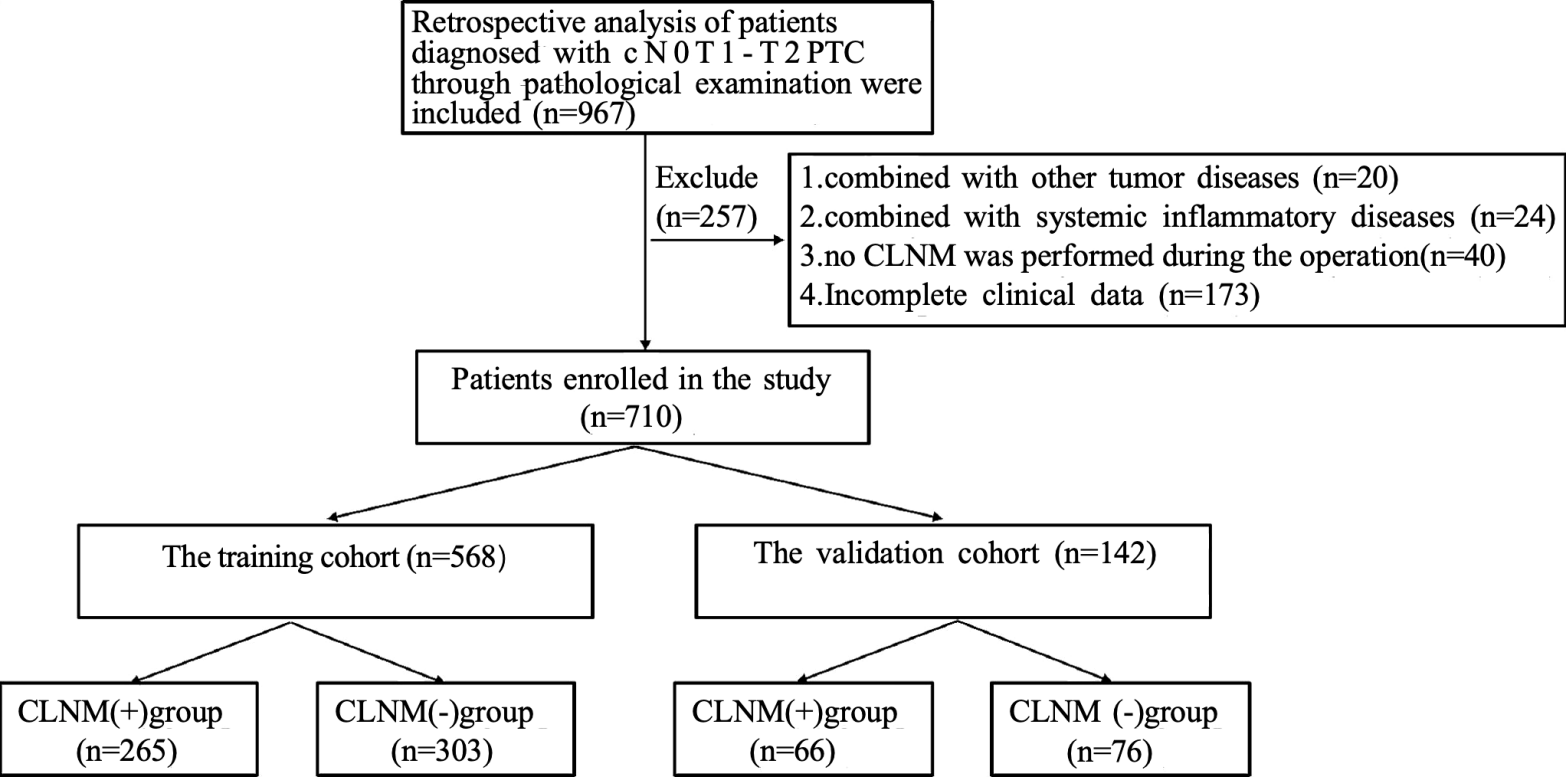
Flowchart of the study.

We reviewed 967 patients diagnosed with cN0T1-T2 PTC, excluded 257 patients based on the flowchart shown in **Figure 2**.** A** total of 710 patients were eventually enrolled. The training cohort included 568 patients (265 with CLNM, 303 without CLNM). The test cohort included 142 patients (66 with CLNM, 76 without CLNM).The participants’ characteristics and their ultrasound, clinical, and laboratory test data were collected.

### 3.2 General information

This study included a total of 710 patients with papillary thyroid carcinoma (PTC), with 971 lesions. The training set consisted of 568 patients and 776 lesions, while the test set consisted of 142 patients and 195 lesions. After removal of 85 overlapping lesions, the final corrected test set comprised 110 lesions. Baseline characteristics were compared between the training and test cohorts, with no significant differences observed (P > 0.05). Baseline traits were compared between CLNM (+) and CLNM (−) groups (**Table 1**).

**Table 1:** Baseline characteristics of the whole cohort grouped by central lymph node metastasis (CLNM) status.

Medical information	Central lymph node metastasis		P value
	CLNM(+) (n=453)	CLNM(-) (n=518)	
Age(year)			
≤55	355 (78.4%)	359 (69.3%)	<0.001**
>55	98 (21.6%)	159 (30.7%)	
Gender			
Famale	271 (59.8%)	397 (76.6%)	<0.001**
Male	182 (40.2%)	121 (23.4%)	
Tumor size(cm)			
≤1.0	287 (63.4%)	417 (80.5%)	<0.001**
>1.0	166 (36.6%)	101 (19.5%)	
Tumor location			
Left	193 (42.6%)	236 (45.6%)	0.144
Right	235 (51.9%)	266 (51.4%)	
Isthmic	25 (5.5%)	16 (3.1%)	
Multifocality			
Solitary tumor	194 (42.8%)	309 (59.7%)	<0.001**
Multifocal tumor	259 (57.2%)	209 (40.3%)	
Laterality			
Unilateral	249 (55%)	391 (75.5%)	<0.001**
Bilateral	204 (45%%)	127 (24.5%)	
Capsular infiltration			
Present	291 (64.2%)	300 (57.9%)	0.04*
Absent	162 (35.8%)	218 (42.1%)	
Component			
Solid	446 (98.5%)	508 (98.1%)	0.807
Cystic	7 (1.5%)	10 (1.9%)	
Echo			
Low echo	445 (98.2%)	509 (98.3%)	1.000
High-level echo/iso-echo	8 (1.8%)	9 (1.7%)	
Shape			
Aspect ratio<1	246 (54.3%)	215 (41.5%)	<0.001**
Aspect ratio > 1	207 (45.7%)	303 (58.5%)	
Boundary			
Unclear	262 (57.8%)	315 (60.8%)	0.36
Clear	191 (42.2%)	203 (39.2%)	
Calcifications			
Absent	184 (40.6%)	266 (51.4%)	<0.001**
Present	269 (59.4%)	252 (48.6%)	
NLR			
≤2.18	313 (69.1%)	377 (72.8%)	0.207
>2.18	140 (30.9%)	141 (27.2%)	
PLR			
≤134.88	287 (63.4%)	320 (61.8%)	0.612
>134.88	166 (36.6%)	198 (38.2%)	
LMR			
≤5.20	185 (40.8%)	165 (31.9%)	0.004**
>5.20	268 (59.1%)	353 (68.1%)	
SII			
≤449.85	248 (54.8%)	316 (61%)	0.049*
>449.85	205 (45.2%)	202 (39%)	
SIRI			
≤0.74	286 (63.1%)	366 (70.7%)	0.013*
>0.74	167 (36.9%)	152 (29.3%)	
FT3 (pmol/L)			
2.76-6.45	451 (99.6%)	517 (99.8%)	0.486
others	2 (0.4%)	1 (0.2%)	
FT4 (pmol/L)			
11.2-23.81	452 (99.8%)	511 (98.6%)	0.052
others	1 (0.2%)	7 (1.4%)	
TSH (μIU/L)			
0.35-5.1	436 (96.2%)	472 (91.1%)	0.001**
others	17 (3.8%)	46 (8.9%)	
Tg (ng/ml)			
≤77	407 (89.8%)	484 (93.4%)	0.042*
>77	46 (10.2%)	34 (6.6%)	
Tg-Ab (IU/ml)			
≤115	433 (95.6%)	492 (95%)	0.658
>115	20 (4.4%)	26 (5%)	
TPO-Ab (IU/ml)			
≤34	411 (90.7%)	489 (94.4%)	0.028*
>34	42 (9.3%)	29 (5.6%)	
TRAb (IU/L)			
≤1.75	424 (93.6%)	487 (94%)	0.788
>1.75	29 (6.4%)	31 (6%)	

Categorical variables are presented as number (%). TSH, thyroid-stimulating hormone; FT3, free triiodothyronine; FT4, free thyroxine; TgAb, thyroglobulin antibody; TPOAb, thyroid peroxidase antibody; TRAb, thyrotropin receptor antibody; Tg, thyroglobulin; CLNM, central lymph node metastasis; NLR, neutrophil-to-lymphocyte ratio; PLR, platelet-to-lymphocyte ratio; LMR, lymphocyte-to-monocyte ratio; SII, systemic immune-inflammation index; SIRI, systemic inflammatory response index.*P<0.05, **P<0.01.

Categorical variables are presented as number (%). CLNM: central lymph node metastasis; NLR: neutrophil-to-lymphocyte ratio; PLR: platelet-to-lymphocyte ratio; LMR: lymphocyte-to-monocyte ratio; SII: systemic immune-inflammation index; SIRI: systemic inflammatory response index.*P<0.05, **P<0.01.

### 3.3 Optimal cut-off values for individual predictors

These cutoffs were used only for descriptive stratification and clinical interpretation; all predictive models were developed using the original continuous variables. The optimal cut-off value of NLR for discriminating CLNM was 2.18, yielding an AUC of 0.581 with a sensitivity of 57.3% and specificity of 56.6%. For PLR, the optimal cut-off was 134.88, corresponding to an AUC of 0.530, sensitivity of 46.4%, and specificity of 58.7%. LMR demonstrated an optimal cut-off of 5.2, with an AUC of 0.585, sensitivity of 55.0%, and specificity of 59.5%. The optimal SII cut-off was 449.85, achieving an AUC of 0.582, sensitivity of 59.2%, and specificity of 59.4%. For SIRI, the optimal cut-off was 0.74, resulting in an AUC of 0.567, sensitivity of 56.9%, and specificity of 54.7%.

Based on these optimal cut-offs, patients were stratified into two groups: low NLR (≤2.18) vs. high NLR (>2.18); low PLR (≤134.88) vs. high PLR (>134.88); low LMR (≤5.2) vs. high LMR (>5.2); low SII (≤449.85) vs. high SII (>449.85); and low SIRI (≤0.74) vs. high SIRI (>0.74).

### 3.4 Feature selection and model development

#### 3.4.1 Within-domain feature screening and single-domain performance

**Figure 3:**
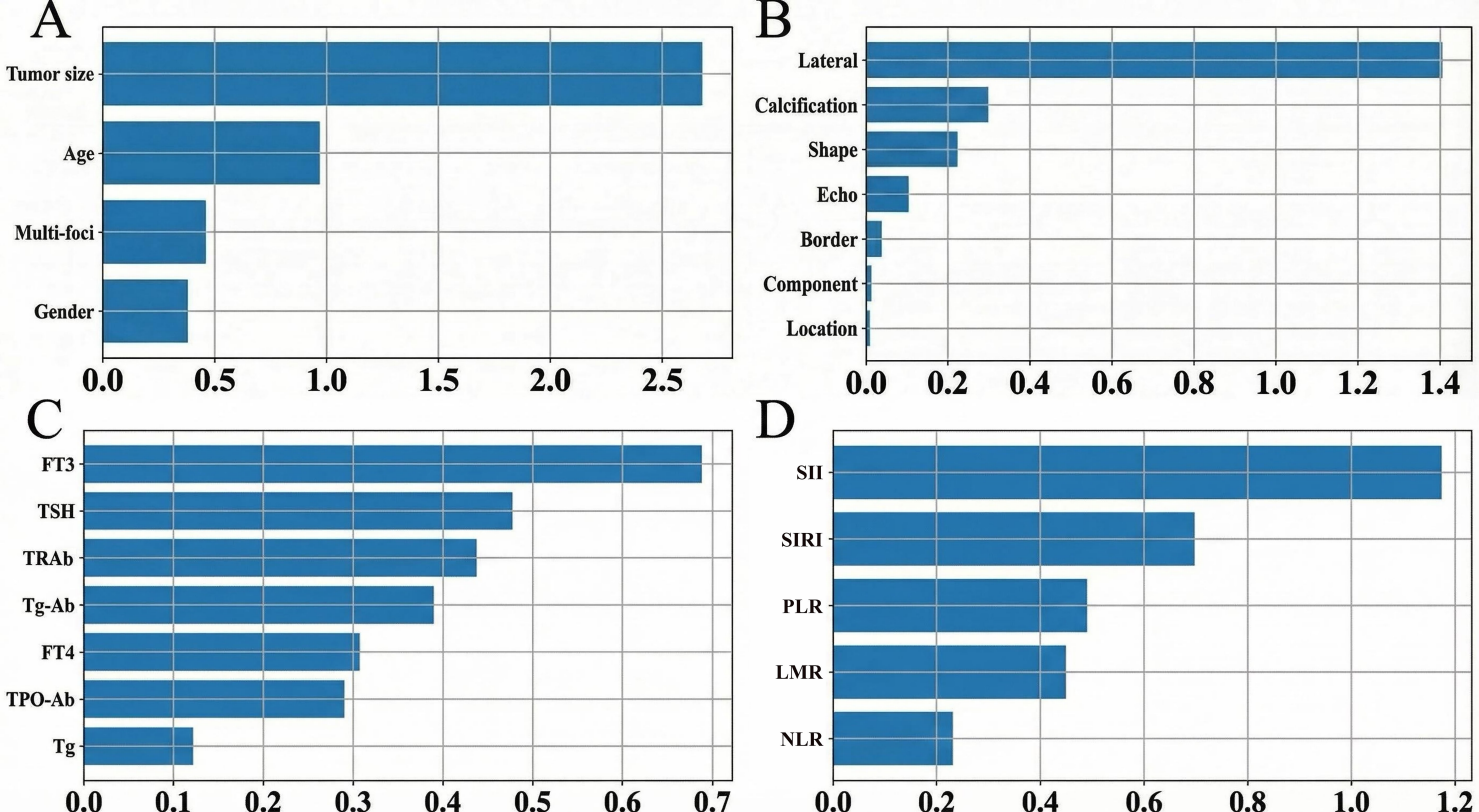
Ranking of feature importance within each domain. **(A)** Pathological features, **(B)** ultrasound features, **(C)** thyroid function indicators, **(D)** inflammatory indicators. Bar length represents the absolute standardized coefficient (|β|).

The within-domain feature importance rankings are detailed in **Figure 3** (pathology features: A; ultrasound characters: B; thyroid function: C; inflammatory indicators: D). We analyzed the influence of inflammatory indicators, ultrasonic characteristics, pathological features and thyroid function on CLNM by drawing ROC curves. The AUCs for ultrasonic characteristics, pathological features, inflammatory indicators and thyroid function to distinguish CLNM were 0.663, 0.715, 0.600 and 0.602, respectively.

#### 3.4.2 Integrated model development and performance comparison

**Figure 4:**
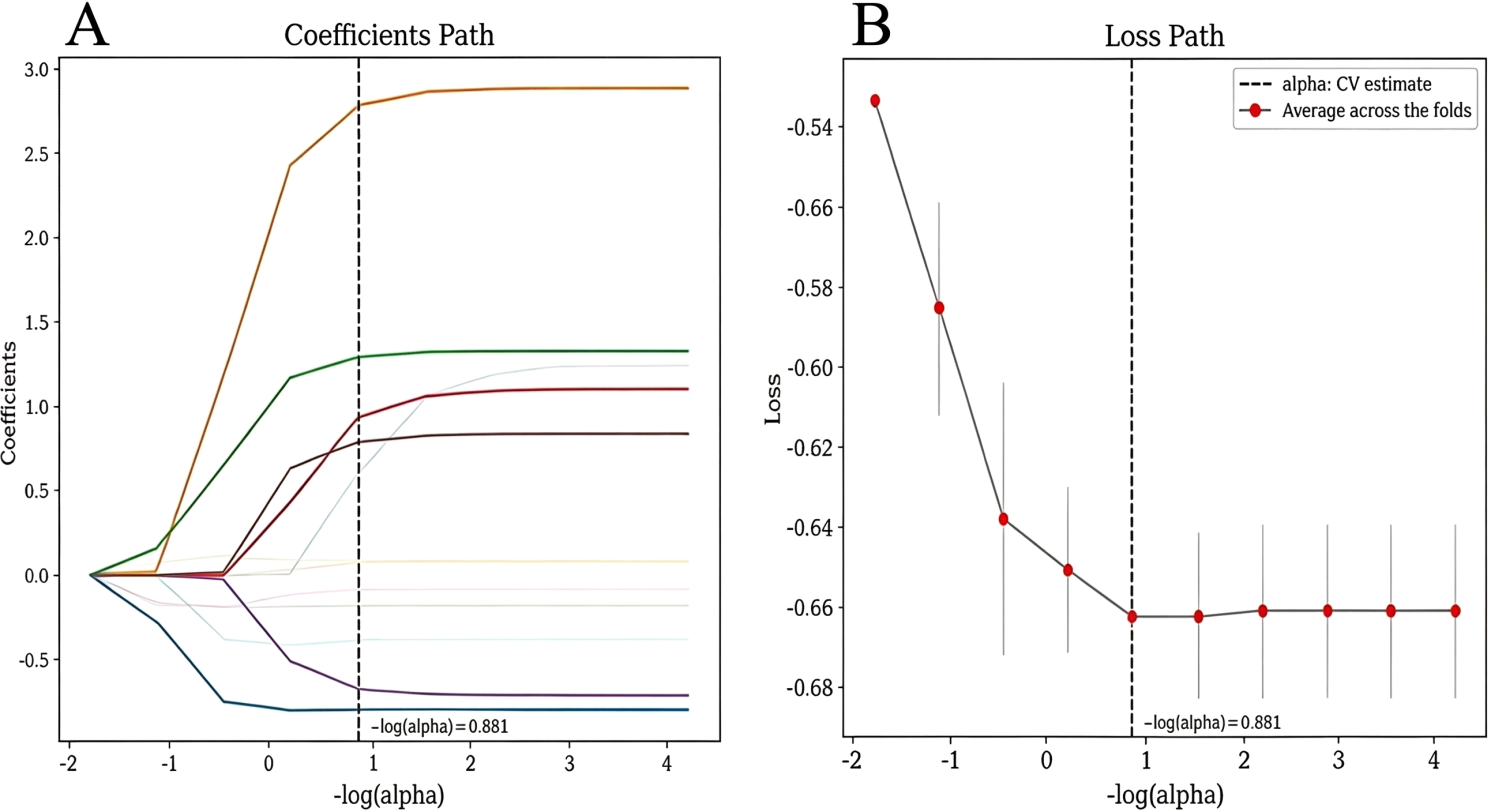
Feature selection using LASSO regression. **(A)** LASSO coefficient profiles of the 6 candidate variables. **(B)** Cross-validation for feature selection; dotted vertical lines indicate the optimal λ values selected by the minimum criteria and the 1-SE rule. LASSO, least absolute shrinkage and selection operator.

LASSO retained six variables: age, tumor size, laterality, FT3, PLR, and SII (**Figure 4**). Multivariable logistic regression identified five independent predictors of CLNM: age, tumor size, laterality, PLR, and SII (all P < 0.05; **Table 2**). FT3, though not statistically significant, was retained as an adjunct variable for its contribution to model performance.

**Table 2:** Results of logistic regression analysis without regularization.

Medical information	β (coefficient)	SE	P	OR	95% CI
Laterality	1.092	0.148	<0.001		
Unilateral				1 (reference)	
Bilateral				2.979	2.227-3.984
Tumor size (cm)	0.085	0.014	<0.001		
≤1.0				1 (reference)	
>1.0				1.088	1.060-1.118
Age (year)	-0.03	0.006	<0.001		
>55				1 (reference)	
≤55				0.971	0.959-0.982
SII	0.001	0	0.005		
≤449.85				1 (reference)1	
>449.85				1.001	1.000-1.002
PLR	-0.006	0.002	0.006		
>134.88				1 (reference)	
≤134.88				0.994	0.990-0.998
FT3 (pmol/L)	0.136	0.105	0.198		
2.76-6.45				1 (reference)1	
others				1.145	0.931-1.408

PLR, platelet-to-lymphocyte; SII, systemic inflammation index.

**Figure 5:**
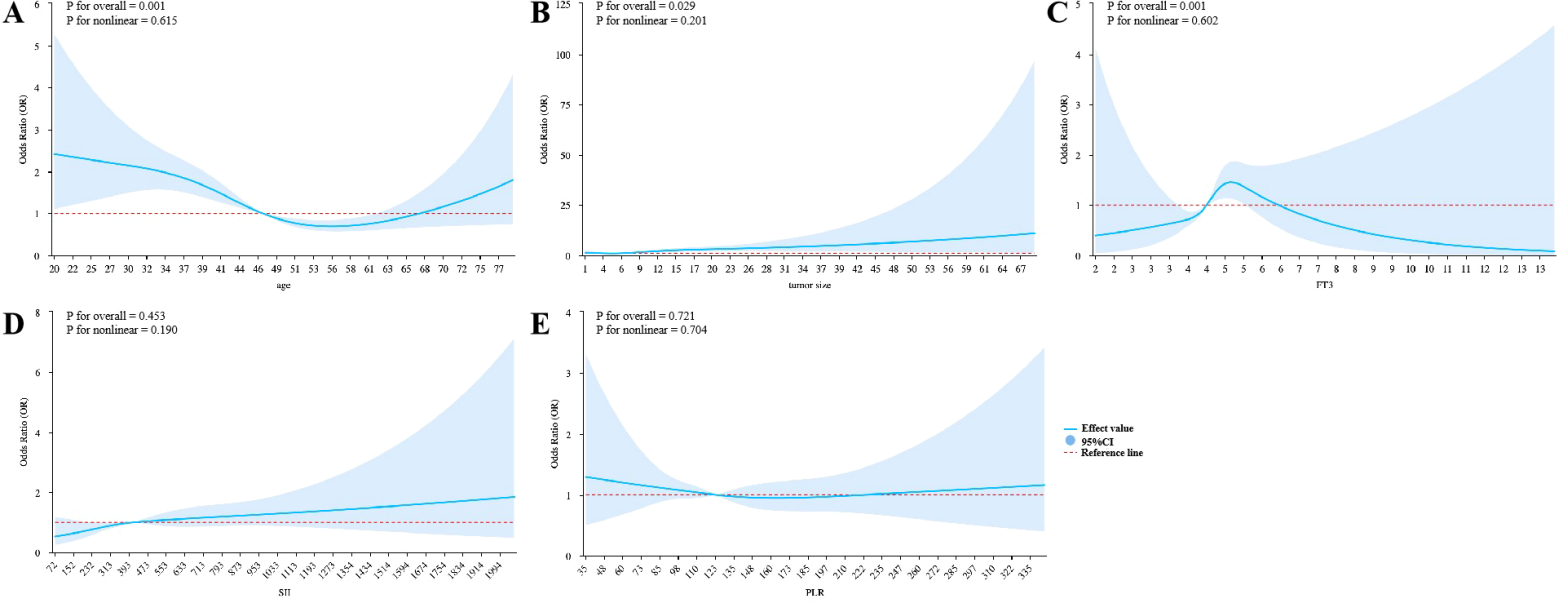
Restricted cubic spline (RCS) plots for continuous variables. The figure illustrates the relationship between continuous variables and central lymph node metastasis (CLNM). Variables analyzed include age **(A)**, tumor size **(B)**, FT3 **(C)**, SII **(D)**, and PLR **(E)**. Each plot shows the nonlinear relationship between the variable and the outcome, with p-values indicating overall significance and nonlinearity. Abbreviations: RCS, restricted cubic spline; PLR, platelet-to-lymphocyte ratio; SII, systemic immune-inflammation index.

RCS analysis confirmed linear relationships for age, tumor size, and FT3 with CLNM risk (overall P < 0.05, nonlinear P > 0.05), with risk thresholds of 55.162 years, 4.434 mm, and 5.07 pmol/L, respectively. No significant associations were observed for SII or PLR (**Figure 5**).

**Figure 6:**
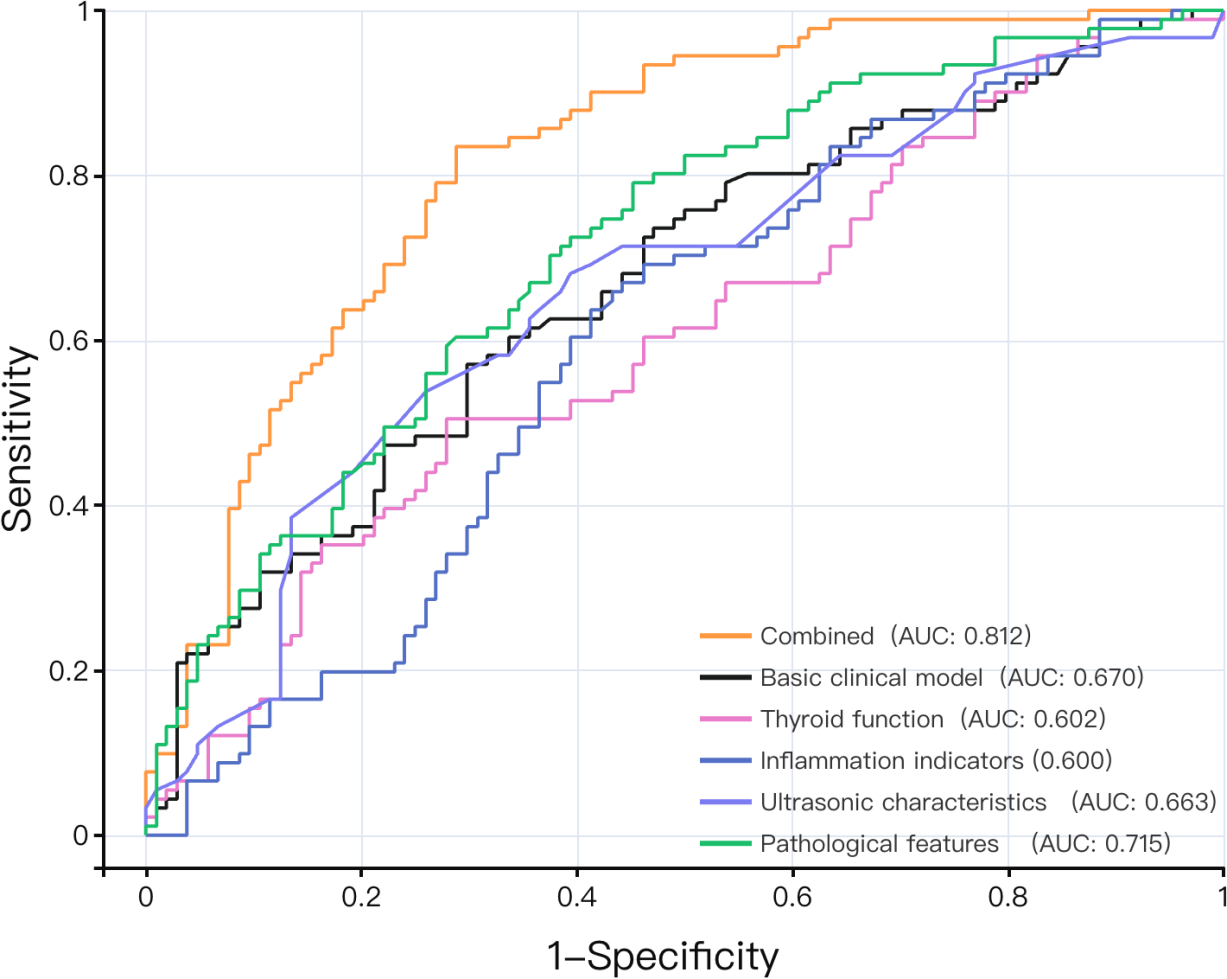
Analysis of the diagnostic value of clinical data. ROC curves were used to evaluate the diagnostic performance of the baseline clinical model, pathological features, ultrasound characteristics, inflammatory indicators, thyroid function indicators, and the combined model for predicting CLNM. ROC analysis was performed on the corrected test set.

Using the six selected features, six machine learning models were evaluated on the test set (**Table 3**). GBDT achieved the best performance (AUC: 0.812; 95% CI: 0.731–0.893), outperforming KNN (0.760), SVM (0.753), and logistic regression (0.750). The combined model AUC of 0.812 exceeded all single-domain models (**Table 4**; **Figure 6**). DeLong test (13) confirmed GBDT significantly outperformed KNN (P = 0.022), SVM (P = 0.001), and logistic regression (P = 0.001), while differences among the latter three were not significant (**Table 5**).

**Table 3:** Predictive performance of ML model.

Parameter		AUC (95% CI)	ACC	SEN (95% CI)	SPE (95% CI)	PPV (95% CI)	NPV (95% CI)	F1-score
KNN	Training cohour	0.872 (0.850, 0.895)	0.769	0.917 (0.884, 0.941)	0.640 (0.593, 0.685)	0.690 (0.648, 0.730)	0.898 (0.859, 0.928)	0.788
	Test cohort	0.760 (0.695, 0.825)	0.692	0.681 (0.580, 0.768)	0.702 (0.608, 0.781)	0.667 (0.566, 0.754)	0.716 (0.622, 0.794)	0.674
SVM	Training cohour	0.707 (0.670, 0.743)	0.649	0.727 (0.678, 0.770)	0.582 (0.534, 0.629)	0.603 (0.557, 0.648)	0.709 (0.658, 0.755)	0.659
	Test cohort	0.753 (0.685, 0.821)	0.692	0.758 (0.661, 0.835)	0.635 (0.539, 0.721)	0.645 (0.551, 0.729)	0.750 (0.650, 0.829)	0.697
LR	Training cohour	0.708 (0.672, 0.745)	0.666	0.539 (0.487, 0.589)	0.778 (0.735, 0.815)	0.679 (0.623, 0.731)	0.685 (0.615, 0.699)	0.601
	Test cohort	0.750 (0.682, 0.819)	0.703	0.802 (0.709, 0.871)	0.615 (0.519, 0.703)	0.646 (0.554, 0.728)	0.780 (0.679, 0.856)	0.716
DT	Training cohour	0.687 (0.651, 0.722)	0.668	0.707 (0.658, 0.752)	0.633 (0.585, 0.678)	0.627 (0.580, 0.673)	0.712 (0.664, 0.756)	0.665
	Test cohort	0.687 (0.616, 0.758)	0.667	0.747 (0.649, 0.825)	0.596 (0.500, 0.685)	0.618 (0.525, 0.704)	0.729 (0.627, 0.812)	0.770
RF	Training cohour	0.691 (0.655, 0.726)	0.648	0.729 (0.681, 0.772)	0.577 (0.529, 0.624)	0.601 (0.555, 0.646)	0.709 (0.659, 0.755)	0.659
	Test cohort	0.690 (0.619, 0.762)	0.667	0.813 (0.721, 0.880)	0.538 (0.443, 0.631)	0.607 (0.518, 0.689)	0.767 (0.658, 0.849)	0.695
GBDT	Training cohour	0.912 (0.892, 0.931)	0.834	0.848 (0.807, 0.881)	0.821 (0.781, 0.855)	0.806 (0.763, 0.842)	0.861 (0.823, 0.891)	0.826
	Test cohort	0.812 (0.731, 0.893)	0.764	0.835 (0.746, 0.897)	0.702 (0.608, 0.781)	0.710 (0.618, 0.788)	0.830 (0.738, 0.894)	0.768

ML, machine learning; DT, decision tree; KNN, K-nearest neighbors; RF, random forest; SVM, support vector machine; GBDT, gradient boosting decision tree; LR, logistic regression; AUC, area under the curve; CI, confidence interval; ACC, accuracy; SEN, sensitivity; SPE, specificity; PPV, positive predictive value; NPV, negative predictive value.

**Table 4:** ROC analysis parameters for the prediction models.

Model	AUC	95%CI	Sensitivity	Specificity	P value
Pathological features	0.715	0.643–0.786	79.1	53.8	<0.001
Ultrasonic characteristics	0.663	0.586–0.740	68.1	60.6	<0.001
Inflammatory indicators	0.600	0.520–0.680	69.2	52.9	<0.001
Thyroid function	0.602	0.523–0.682	50.5	71.2	<0.001
Basic clinical model	0.670	0.594–0.746	57.1	69.2	<0.001
Combined	0.812	0.731–0.893	83.5	70.2	<0.001

AUC Area under the curve.

**Table 5:** Performance comparison of different machine learning models and results of DeLong test.

Model	AUC (95% CI)	The P-value compared with GBDT
GBDT	0.812 (0.731, 0.893)	–
KNN	0.760 (0.695, 0.825)	0.022*
SVM	0.753 (0.685, 0.821)	0.001*
LR	0.750 (0.682, 0.819)	0.001*

GBDT, Gradient Boosting Tree; KNN, K-nearest neighbors; SVM, support vector machine; LR: logistic regression, *P < 0.05.

#### 3.4.3 Comparison with baseline clinical model

**Figure 7:**
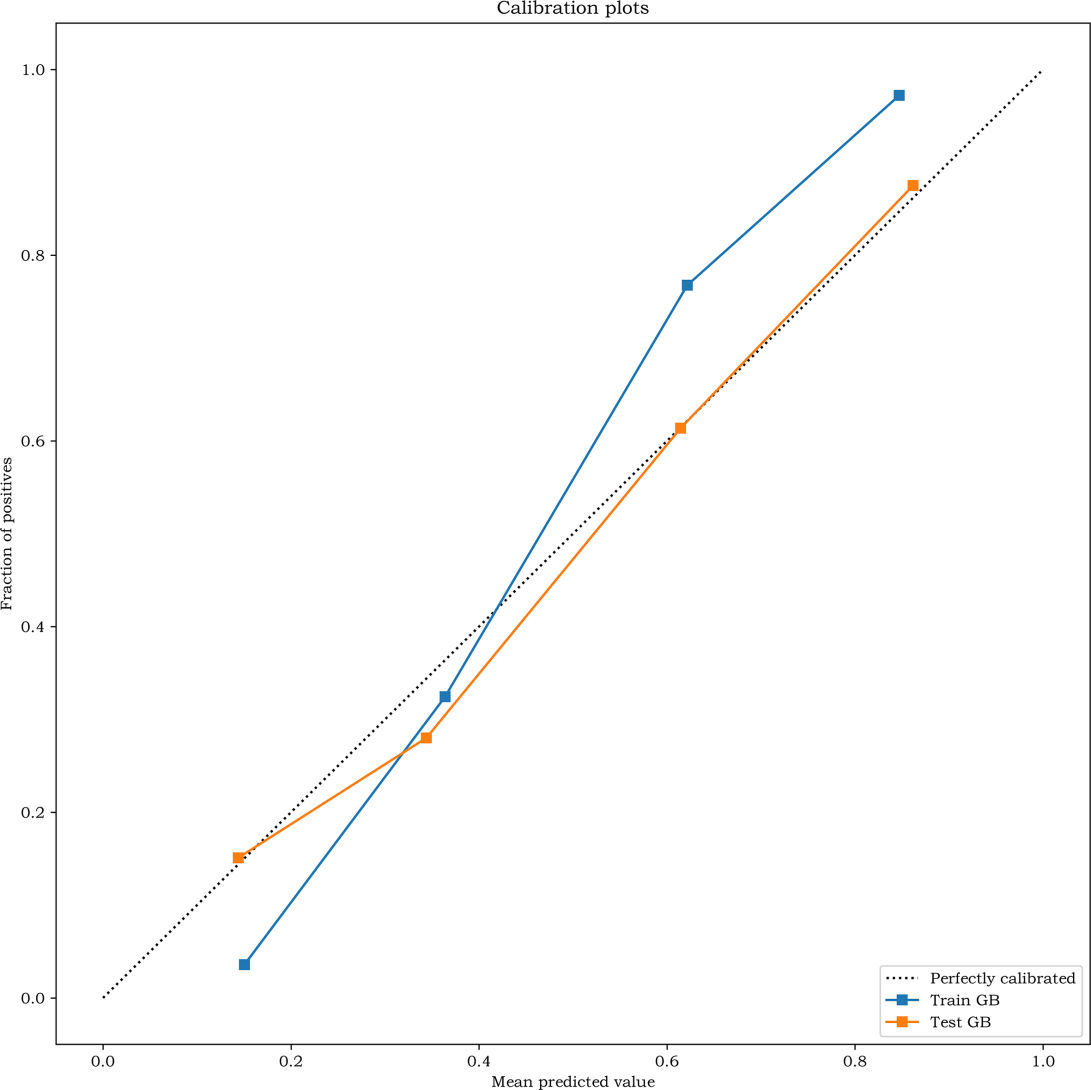
Calibration curves of the prediction model in the training and the corrected test set.

A simple baseline model (age, tumor size, laterality) achieved an AUC of 0.670 in the test cohort (Brier 0.2270, intercept 0.0272, slope 0.8968). The combined model showed improved discrimination (AUC: 0.812 vs. 0.67; +0.142) and better calibration (Brier: 0.17 vs. 0.23; intercept: 0.05 vs. 0.03; slope: 1.45 vs. 0.90), confirming the incremental value of inflammatory markers and machine learning (**Figure 7**).

### 3.5 Model performance

The calibration curve showed good consistency (**Figure 7**). The model achieved an AUC of 0.919 (95% CI: 0.900–0.937) in the training cohort and 0.830 (95% CI: 0.773–0.887) in the test cohort, demonstrating good discrimination.

#### 3.5.1 Model performance on the corrected test set

After removing 85 overlapping lesions, the final testing set comprised 110 lesions with no training-set overlap. On this corrected set, the model achieved an AUC of 0.812 (95% CI: 0.731–0.893), with 76.4% accuracy, 83.5% sensitivity, and 70.2% specificity.

#### 3.5.2 Ablation analysis of FT3

To empirically validate the contribution of FT3, we performed an ablation analysis comparing model performance with and without FT3 on the corrected test set (n=110). The model without FT3 achieved an AUC of 0.731 (95% CI: 0.656–0.805), a calibration slope of 0.85, and a DCA net benefit of 0.88 at a threshold of 0.1. Adding FT3 improved model performance, with the full model achieving an AUC of 0.812 (95% CI: 0.731–0.893), a calibration slope of 1.45, and a DCA net benefit of 0.93 at the same threshold. These results demonstrate that FT3 contributes meaningfully to model performance despite its non-significant p-value in multivariable analysis, ruling out potential overfitting.

#### 3.5.3 Patient-level sensitivity analysis

To address within-patient clustering, we constructed an independent patient-level test set comprising 100 patients with solitary lesions (49 CLNM-positive, 51 negative) from the corrected testing set. On this set, the model achieved an AUC of 0.804 (95% CI: 0.718–0.890), with 73.5% sensitivity and 76.5% specificity. This result is consistent with the primary lesion-level analysis (AUC 0.812), indicating no substantial bias from within-patient clustering.

#### 3.5.4 External validation

The model was evaluated on an independent external cohort of 50 patients. It achieved an AUC of 0.800 (95% CI: 0.664–0.936), with an accuracy of 78%, sensitivity of 87.5%, and specificity of 69.2%. This result is consistent with the internal validation (AUC 0.812), demonstrating good generalizability of the model (**Supplementary Table S4, Supplementary Figure S1**).

### 3.6 Decision curve analysis

**Figure 8:**
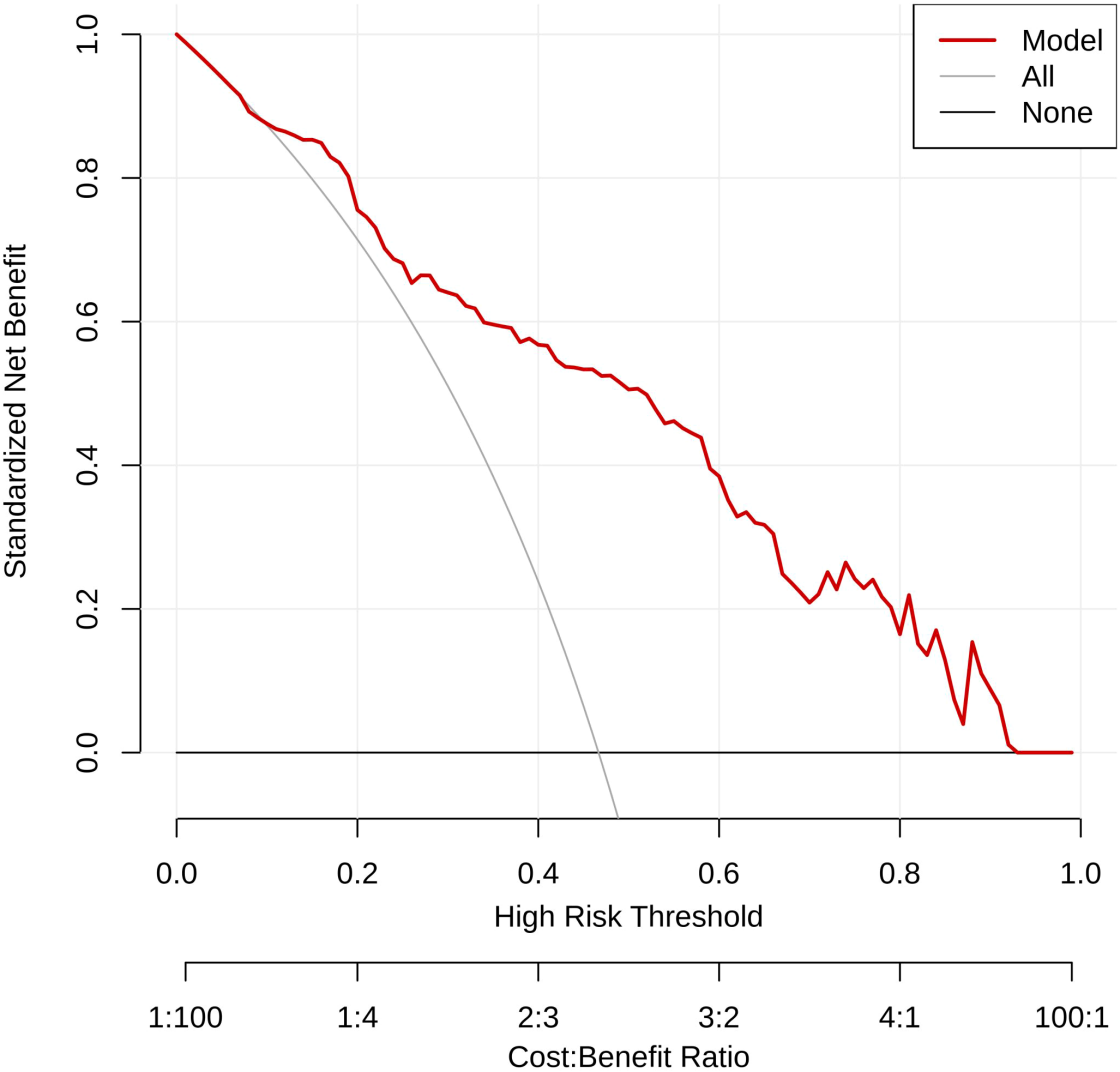
Decision curve analysis for the GBDT model in the corrected test cohort.

As shown in **Figure 8**, the combined model provided positive net benefit across a wide threshold range (0%–85%), with peak performance in the clinically relevant 10%–50% interval. Within this range, the model substantially outperformed both treat-all and treat-none strategies.

Clinical interpretation: When predicted CLNM risk is 10%–50%, the model can guide prophylactic central lymph node dissection by balancing overtreatment and undertreatment. For example, at a 20% threshold, net benefit was 0.30, comparable to treat-all (0.31) and superior to treat-none (0). These findings support the model’s clinical utility in individualizing surgical decisions.

The x-axis represents the predicted probability of central lymph node metastasis (CLNM), and the y-axis represents the observed proportion. The diagonal dotted line indicates perfect calibration, while the solid line represents the model’s performance. A closer fit to the diagonal line indicates better prediction accuracy.

### 3.7 Explanation of the ML model with the SHAP method

**Figure 9:**
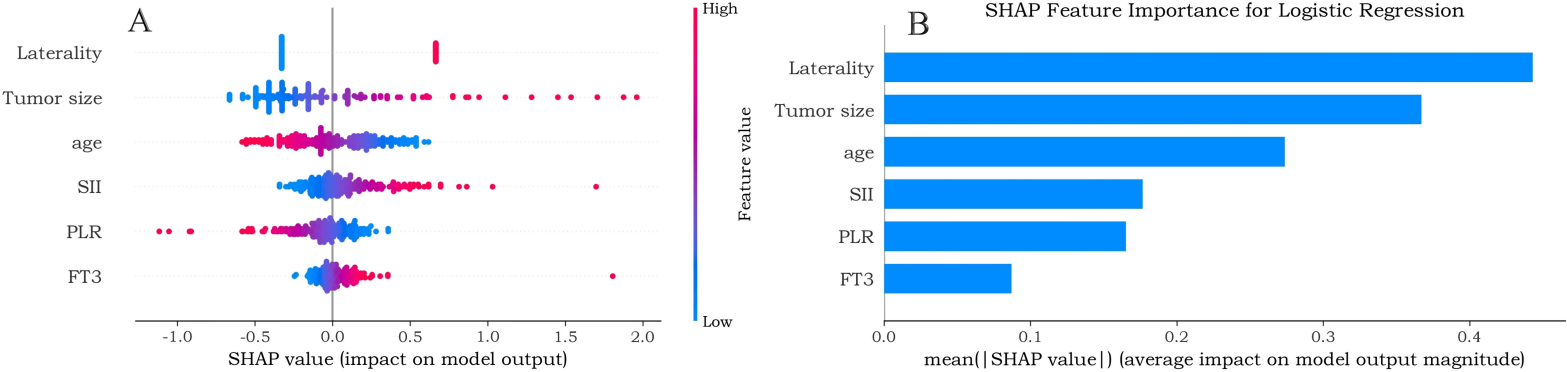
SHAP summary plots for the prediction model of central lymph node metastasis. **(A)** Beeswarm plot showing the distribution and direction of SHAP values for each feature. **(B)** Bar plot illustrating global feature importance based on mean absolute SHAP values.

The SHAP method was employed to quantify the contribution of each feature to the model’s predictions. The beeswarm plot (**Figure 9A**) illustrates feature directionality: higher tumor size, SII, and FT3 correlate with increased CLNM probability, while lower age and PLR correlate inversely. The SHAP summary bar chart (**Figure 9B**) illustrates global feature importance, ranking features in descending order of mean absolute SHAP values: laterality, tumor size, age, SII, PLR, and FT3. At the local level, SHAP values enable case-specific interpretation, enhancing clinical transparency and supporting individualized decision-making.

## 4 Discussion

The global incidence of thyroid cancer has risen notably in recent years, with papillary thyroid carcinoma (PTC) representing the predominant pathological subtype (2). Lymphatic dissemination is the most common metastatic route in PTC, typically progressing from the central compartment lymph nodes to the lateral cervical nodes (14). Central lymph node metastasis (CLNM) is a frequent and prognostically significant determinant in PTC. However, conventional imaging modalities such as ultrasound and contrast-enhanced CT exhibit limited sensitivity in the preoperative detection of nodal involvement (15). For patients with T1–T2 stage, clinically node-negative (cN0) PTC, the decision to perform prophylactic central neck dissection (pCND) remains a persistent clinical dilemma. Current guidelines predominantly rely on a limited set of pathological features—including tumor size and extrathyroidal extension—for risk stratification (16–18). The predictive accuracy of these parameters is modest, often resulting in either overtreatment or undertreatment. Machine learning has recently emerged as a promising approach for developing predictive models in oncology. Several studies have attempted to construct models for predicting lymph node metastasis in PTC using ultrasound radiomics or conventional clinical variables. Nevertheless, most existing models are constrained by two critical limitations: first, they do not systematically incorporate biologically relevant indicators reflecting the tumor microenvironment or systemic immune status, such as systemic inflammatory indices; second, they often function as “black-box” systems, lacking clinical interpretability, which substantially limits their acceptance and practical utility in routine clinical practice (19, 20). To address these limitations, we developed a multimodal approach guided by clinical domain knowledge. This approach integrates systematic data fusion with a lightweight modeling strategy designed for clinical practice. Several unique features distinguish our work from previous studies. First, we integrated endocrine biomarkers (FT3) and inflammatory indices (SII, PLR). This addresses a gap in prior studies that focused mainly on clinical and ultrasound features. Second, we applied rigorous data leakage correction. We identified and removed overlapping lesions between the training and test sets, ensuring unbiased model evaluation. Third, we provided comprehensive model interpretability using SHAP analysis. This revealed global feature importance and enabled individualized prediction explanations. Fourth, we conducted multiple robust sensitivity analyses. These included patient-level clustering effect assessment and external validation on an independent temporal cohort (n=50, AUC 0.800). Together, these analyses further support the generalizability of our model. As a result, a predictive model was developed that demonstrates high performance, strong interpretability, and structural simplicity.

Tumor invasiveness is influenced by both the tumor microenvironment and pathological characteristics, limiting the diagnostic performance of ultrasound alone. To address this, we developed a multimodal framework that integrates ultrasound characters, pathological features, inflammatory indicators, and thyroid function parameters. Unlike conventional feature aggregation, our approach employs a hierarchical, domain-guided selection strategy using an “intra-domain screening, cross-domain integration” workflow. Within four clinical domains, key features were selected via L2−regularized logistic regression. This design preserves clinical specificity, enhances feature robustness through noise reduction, and improves interpretability compared to radiomics or deep-learning “black-box” models. Moreover, by incorporating multimodal data, our framework reduces dependence on high-quality imaging and standardized annotations, increasing its practicality and generalizability in clinical practice. This study successfully developed and validated six interpretable machine learning models with a prediction performance (AUC = 0.812). Our research results are consistent with those of (Qiao et al., 2024) (21), who included a similar feature-based nomogram model in 1, 392 PTC patients and also achieved an AUC value of 0.809.

To assess the relative performance of our model, we compared it with recently reported state-of-the-art (SOTA) models for predicting cervical lymph node metastasis in papillary thyroid carcinoma. On the corrected test set after removing overlapping lesions, our GBDT multimodal model achieved an AUC of 0.812 (95% CI: 0.731–0.893), which is higher than the pooled AUC of 0.762 from a meta-analysis of 107 studies by Liu et al. (22). Our model also outperforms the nomogram models by Deng et al. (20) (AUC 0.765) and Wang et al. (23) (AUC 0.795), and shows comparable or slightly superior performance to the nomogram by Qiao et al. (21) (AUC 0.809) and the deep learning model by Zhang et al. (24) (AUC 0.81). This improvement may be attributed to the inclusion of endocrine (FT3) and inflammatory (SII, PLR) biomarkers and the use of gradient boosting. For cN0 PTC patients, our model achieved a comparable AUC to the deep learning model by Zhang et al. (24) (AUC 0.81) but uses simpler, more accessible variables. Although the radiomic nomogram by Tong et al. (25) achieved a higher AUC (0.914), it requires specialized feature extraction, whereas our model uses routinely collected variables, offering better clinical accessibility.

A key methodological consideration is the potential non-independence of multiple lesions within the same patient due to shared host factors. To assess clustering effects, we performed a patient-level sensitivity analysis using 100 solitary-lesion patients from the corrected testing set. The model achieved an AUC of 0.804 (95% CI: 0.718–0.890) on this set, comparable to the primary lesion-level analysis (AUC 0.812). The minimal difference and overlapping CIs indicate that within-patient clustering does not materially bias our findings. An AUC of 0.804 remains clinically meaningful and consistent with published models (19, 21), supporting model robustness.

The traditional prognostic assessment methods, such as TNM staging and clinical pathological features, although playing a crucial role in clinical applications, have limitations in revealing the biological characteristics and immune microenvironment of tumors, thereby highlighting the complementary value of serum indicators (26). Studies have shown that the combined inflammatory indicators calculated from the counts of neutrophils, lymphocytes, monocytes, and platelets in peripheral blood, namely the lymphocyte-related inflammatory index, can reflect the state of infiltrating inflammatory cells in tumor tissues. As an indirect indicator reflecting the host’s immune status, it is helpful for assisting in the diagnosis and prognosis assessment of cancer patients (12, 27, 28). These indicators have been proven to be effective factors for predicting the prognosis and tumor progression of cancer patients (12, 27). Some studies have confirmed that LMR, PLR, NLR, and SIRI are predictive factors for CLNM and LLNM in PTC patients (28). The preoperative levels of NLR, PLR, and SII in peripheral blood can reflect the biological characteristics of PTC, and higher NLR, PLR, and SII values are closely related to poorer pathological histological features and more aggressive clinical biological behaviors (29). Zhang et al. (3)found that SII level were independently associated with central lymph node metastasis in cN0PTC patients. The SII-based model yielded a significant result in the PTC cohort, with an AUC of 0.814. Consistent with existing evidence (3, 21), our results identify SII > 449.85 (OR = 1.001, P = 0.005) and PLR<134.88 (OR = 0.994, P = 0.006) as independently risk factors for CLNM.

The TNM staging system uses 55 years as the age cutoff in thyroid cancer. Age is a significant risk factor for CLNM and a marker of poor prognosis in patients with papillary thyroid microcarcinoma (PTMC) (30). Our study further demonstrated that patients younger than 55 years had a higher incidence of CLNM compared to older patients, with age serving as an independent predictor of CLNM (OR = 0.971, P = 0.000). Therefore, preoperative central lymph node assessment should be prioritized in young PTMC patients (31).

Tumor diameter critically influences prognostic stratification. The TNM system recognizes that lesions>1 cm exhibit progressively greater invasive potential. Consistent with this, our study found tumor size>1 cm to be significantly associated with a higher rate of CLNM in PTC patients (OR = 1.088, P = 0.000). We therefore strongly advise meticulous central lymph node dissection for these patients. In other similar studies involving 400 cases of PTC, the rate of CLNM (cervical lymph node metastasis) in PTC patients with tumors>1 cm was significantly higher than that in patients with tumors < 1 cm (32).

Bilaterality is a significant risk factor for CLNM in cN0 papillary thyroid carcinoma. A large-scale meta-analysis (31 studies, 37, 355 patients) established bilaterality as a significant correlate of CLNM in cN0 papillary thyroid carcinoma (OR = 1.52, 95% CI: 1.31–1.77, P < 0.001) (33). Furthermore, a separate cohort study of 1, 304 patients independently identified bilaterality as an independent predictor of CLNM, further corroborating this association (34). Furthermore, bilateral involvement was confirmed as an independent risk factor for CLNM in our analysis (OR = 2.979, P = 0.000).

While elevated serum FT3 has been linked to lymph node metastasis in PTC, its specific association with central LNM (CLNM) remains undefined. Wang et al. (35) demonstrated a positive correlation between FT3 and LNM (P < 0.001), another study identified FT3 as an independent risk factor for LNM (OR = 1.448, P = 0.039) (36). In our multivariable analysis, FT3 was not independently associated with CLNM (OR = 1.145, P = 0.198). Yet, adding FT3 to the multimodal model improved predictive performance, increasing the AUC from 0.731 to 0.812. Ablation analysis confirmed that FT3 contributed meaningfully to model performance, with improvements in AUC, calibration, and DCA net benefit, ruling out potential overfitting. The calibration slope of the model with FT3 was 1.45, indicating slight overdispersion of predicted probabilities. This may be attributed to the limited sample size of the corrected test set (n=110). This supports the methodological principle that retaining clinically relevant variables can enhance model utility beyond statistical significance alone (37). This seemingly contradictory phenomenon may be due to the fact that FT3 is not a direct driving factor, but as a sensitive system biomarker that reflects the interaction between the thyroid hormone system activity and the tumor microenvironment. According to Diessl et al. (38), higher FT3 levels correlate with worse clinical outcomes in advanced differentiated thyroid cancer. This has also been supported by preclinical research, thyroid hormone affects cancer cells through a variety of non-genetic pathways (including activating cell membrane receptor integrin αvβ3) (39), namely that thyroid hormone signaling can affect tumor behavior, highlighting the impact of even subtle functional changes on the tumor microenvironment (40).The underlying mechanism thus suggests that FT3 may not act as a direct risk factor, but rather as a systemic biomarker that reflects the body’s metabolic state and interacts with the tumor microenvironment, providing supplementary information beyond the ultrasonic and pathological features of the model. These findings collectively suggest a potential role of thyroid hormone levels in tumor progression. The systemic inflammation indicators (such as SII and PLR) included in this study essentially reflect the state of systemic immune inflammation, and FT3 may synergize or interact with it from the perspective of endocrine regulation to jointly portray a “hormonal-inflammatory” co-regulatory microenvironment that is more conducive to transfer.

Decision curve analysis further supports the model’s clinical applicability, demonstrating positive net benefit across a wide threshold range (0%–85%) and strong performance in the clinically challenging 10%–50% risk interval. Within this range, the model can guide prophylactic central lymph node dissection by balancing overtreatment and undertreatment, offering a personalized approach for cN0 PTC patients.

Machine learning is often regarded as a “black-box” technology due to its inherent lack of interpretability, which significantly hinders its clinical adoption, given that transparent and explainable decision-making is essential in medical practice (41). This study addressed this challenge by employing the SHAP method, which provides both global and individual patient-specific explanations for model predictions. By using the routinely collected clinical data as input, our model maintained its practicality and was able to integrate into the existing workflow, thereby improving clinical decision-making for patients.

This study also has several limitations. Firstly, it is a retrospective study, which may lead to selection bias. Secondly, this study is a single-center study with preliminary external validation on a limited sample (n=50). Larger-scale multi-center prospective studies are needed to further confirm the generalizability of our model. Thirdly, the ultrasound examination results are largely dependent on the diagnostic experience of the operator, and subjective factors may still affect the data. Fourth, although we validated our model on an independent patient cohort to address sample overlap, the inherent clustering of multiple lesions within patients remains a methodological consideration for lesion-level analyses. Future studies employing hierarchical models may further strengthen generalizability. Fifth, while we compared our method with classical interpretable models (KNN and SVM), future studies should include comparisons with more recent approaches such as XGBoost and deep learning-based models.

In summary, this study identified five independent predictors for central lymph node metastasis (CLNM) in patients with cN0 papillary thyroid carcinoma: bilateral, tumor size > 1.0 cm, age ≤ 55 years, SII > 449.85, and PLR ≤ 134.88. FT3 was incorporated as an adjunct predictor to enhance model performance, reflecting a potential endocrine–inflammatory synergy within a “hormone–inflammation” regulatory microenvironment. Importantly, SHAP-based interpretability analysis elucidated the model’s decision logic, overcoming the “black-box” limitation. The model not only demonstrates strong predictive performance, aiding clinicians in accurate preoperative CLNM assessment to avoid under- or overtreatment, but also offers a novel strategy for personalized management of cN0 T1–T2 PTC patients.

## Data Availability

The original contributions presented in the study are included in the article/**Supplementary Material**. Further inquiries can be directed to the corresponding author.

## Supplementary material

Supplementary Figure S1 ROC curve for external validation. ROC curve of the GBDT model on the external validation cohort (n=50).
