# Supplementary figures and images for: An interpretable machine learning model for predicting central lymph node metastasis in cN0 T1–T2 papillary thyroid carcinoma: a retrospective study
Source: Front Endocrinol (Lausanne). 2026 Apr 27;17:1803663. doi: 10.3389/fendo.2026.1803663 (PMC13158074; doi:10.3389/fendo.2026.1803663)

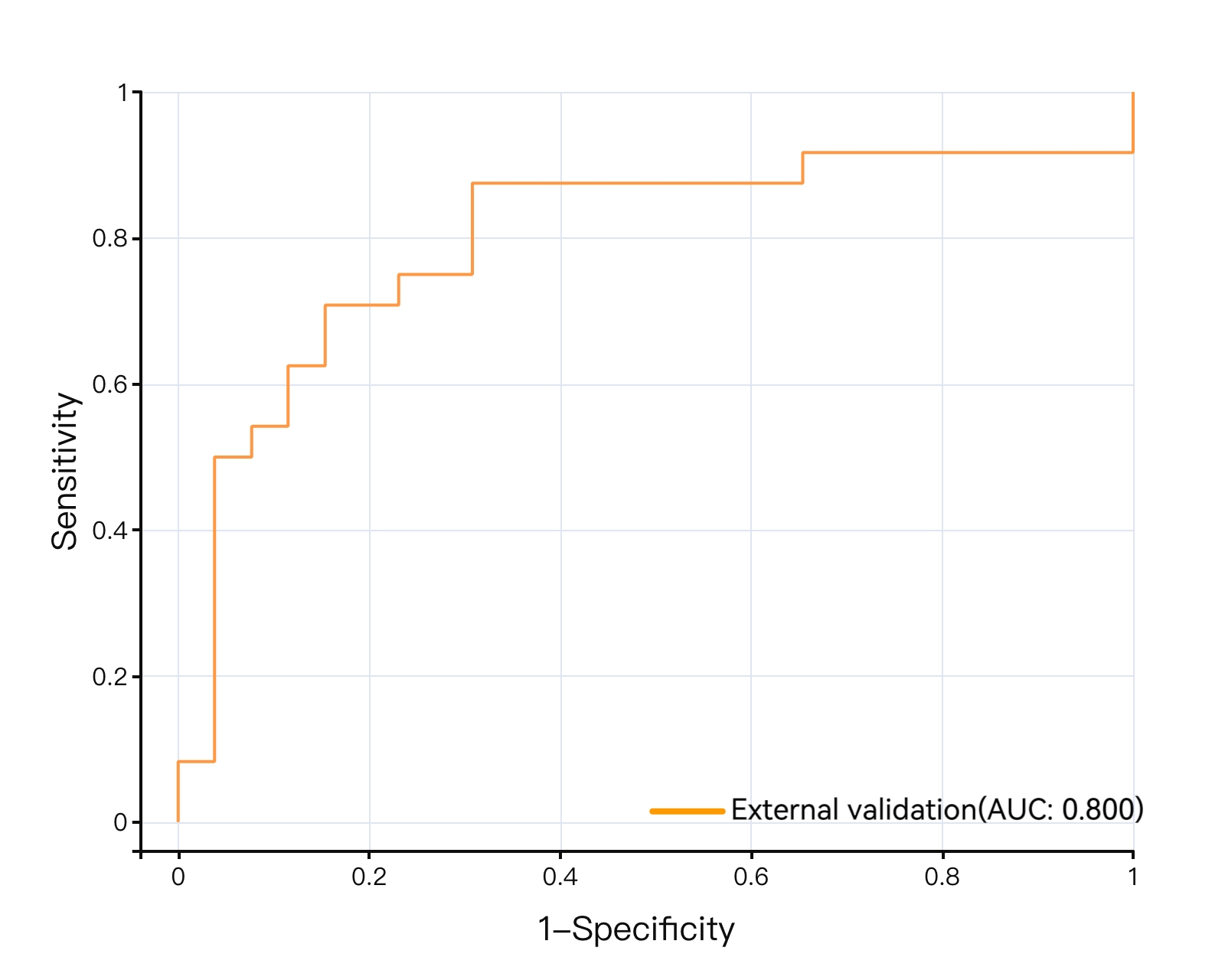

Supplement: Supplementary Figure S1 — ROC curve for external validation. ROC curve of the GBDT model on the external validation cohort (n=50). [file Image1.jpeg]
