# Supplementary material for: An interpretable machine learning model for predicting central lymph node metastasis in cN0 T1–T2 papillary thyroid carcinoma: a retrospective study
Source: Front Endocrinol (Lausanne). 2026 Apr 27;17:1803663. doi: 10.3389/fendo.2026.1803663 (PMC13158074; doi:10.3389/fendo.2026.1803663)
Supplement: Supplementary file 2 [file Table1.docx]

Supplementary Table S1. Patient-Level Sensitivity Analysis

| Metric | Value (95% CI) |
| --- | --- |
| AUC | 0.804（0.718-0.890） |
| Sensitivity | 73.5% |
| Specificity | 76.5% |

This patient-level sensitivity analysis included 100 independent patients with solitary lesions (49 CLNM-positive, 51 negative) from the corrected testing set, with no overlap with the training set. Each patient contributed a single lesion, eliminating within-patient clustering.
