# Supplementary material for: An interpretable machine learning model for predicting central lymph node metastasis in cN0 T1–T2 papillary thyroid carcinoma: a retrospective study
Source: Front Endocrinol (Lausanne). 2026 Apr 27;17:1803663. doi: 10.3389/fendo.2026.1803663 (PMC13158074; doi:10.3389/fendo.2026.1803663)
Supplement: Supplementary file 3 [file Table2.docx]

| Model | AUC (95%CI) | Calibration Slope | DCA Net Benefit (threshold 0.1) |
| --- | --- | --- | --- |
| Without FT3 | 0.731(0.656,0.805) | 0.85 | 0.88 |
| With FT3 | 0.812 (0.731,0.893) | 1.44 | 0.93 |

Supplementary Table S2. Ablation Analysis of FT3
