# Supplementary material for: An interpretable machine learning model for predicting central lymph node metastasis in cN0 T1–T2 papillary thyroid carcinoma: a retrospective study
Source: Front Endocrinol (Lausanne). 2026 Apr 27;17:1803663. doi: 10.3389/fendo.2026.1803663 (PMC13158074; doi:10.3389/fendo.2026.1803663)
Supplement: Supplementary file 4 [file Table3.docx]

Supplementary Table S3. Ablation analysis of top feature count (K) per domain

| K | Pre-screened Features | Final Features (after LASSO) | AUC (95% CI) |
| --- | --- | --- | --- |
| 1 | 4 | 2 | 0.703(0.630,0.776) |
| 2 | 8 | 4 | 0.705(0.631,0.778) |
| 3 | 12 | 6 | 0.812(0.731,0.893) |
| 4 | 16 | 7 | 0.761(0.693,0.828) |
