# Supplementary material for: An interpretable machine learning model for predicting central lymph node metastasis in cN0 T1–T2 papillary thyroid carcinoma: a retrospective study
Source: Front Endocrinol (Lausanne). 2026 Apr 27;17:1803663. doi: 10.3389/fendo.2026.1803663 (PMC13158074; doi:10.3389/fendo.2026.1803663)
Supplement: Supplementary file 5 [file Table4.docx]

Supplementary Table S4. External validation performance

| Metric | Value (95% CI) |
| --- | --- |
| AUC | 0.800 (0.664–0.936) |
| ACC | 0.780 |
| SEN | 0.875 (0.690-0.957) |
| SPE | 0.692 (0.500-0.835) |
| PPV | 0.724 (0.543-0.853) |
| NPV | 0.857 (0.654-0.950) |
| F1 Score | 0.780 |

AUC, area under the curve; CI, confidence interval; ACC, accuracy; SEN, sensitivity; SPE, specificity; PPV, positive predictive value; NPV, negative predictive value.
