# Supplementary material for: An interpretable machine learning model for predicting central lymph node metastasis in cN0 T1–T2 papillary thyroid carcinoma: a retrospective study
Source: Front Endocrinol (Lausanne). 2026 Apr 27;17:1803663. doi: 10.3389/fendo.2026.1803663 (PMC13158074; doi:10.3389/fendo.2026.1803663)
Supplement: Supplementary file 6 [file Table5.docx]

| Strategy | Final Features | AUC (95% CI) |
| --- | --- | --- |
| No screening (12 features) | 12 | 0.756(0.713,0.799) |
| Single-stage screening (23 → LASSO) | 11 | 0.764(0.697,0.830) |
| Two-stage screening (ours) | 6 | 0.812(0.731,0.893) |

Supplementary Table S5. Ablation analysis of feature selection strategies
